# Complementary alternative medicine use among patients with type 2 diabetes mellitus in the primary care setting: a cross-sectional study in Malaysia

**DOI:** 10.1186/1472-6882-13-148

**Published:** 2013-06-26

**Authors:** Siew Mooi Ching, Zainul Amiruddin Zakaria, Fuziah Paimin, Mehrdad Jalalian

**Affiliations:** 1 Department of Family Medicine, Faculty of Medicine and Health Sciences, Universiti Putra Malaysia, Serdang, Selangor 43400, Malaysia; 2 Department of Biomedical Science, Faculty of Medicine and Health Sciences, Universiti Putra Malaysia, Serdang, Selangor 43400, Malaysia; 3 Family medicine specialist, Klinik Kesihatan Salak, Sepang, Malaysia; 4 M.D., Mashhad, Iran

**Keywords:** Complementary alternative medicine, Diabetes mellitus, Primary care, Malaysia, Prevalence

## Abstract

**Background:**

Limited study on the use of complementary alternative medicine (CAM) among patients with diabetes mellitus (DM), particularly in primary -care settings. This study seeks to understand the prevalence, types, expenditures, attitudes, beliefs, and perceptions of CAM use among patients with DM visiting outpatient primary care clinics.

**Methods:**

This is a descriptive, cross-sectional study of 240 diabetic patients. CAM is defined as a group of diverse medical and healthcare systems, practices, and products that are not generally considered part of conventional Western medicine. Data analysis was done using SPSS v. 19 and multiple logistic regressions were used to identify predictors of CAM use.

**Results:**

The prevalence of CAM use was 62.5 percent. Female were 1.8 times more likely than male in using CAM. Malays (75%) were the most frequent users, followed Indians (18%) and Chinese (6%). Biological therapy (50.0%) were the most widely used, followed by manipulative-body based systems (9.2%), energy system (8.8%), alternative medicine systems (4.6%) and mind-body system (1.7%). In biological therapy, a total of 30.4 percent, 24.2 percent, 13.3 percent, and 7.9 percent of diabetic patients consumed bitter gourd (*Momordica Charantia*), followed by Misai Kucing (*Orthosiphon Stamineus Benth*), garlic (*Allium Sativum*), and Sabah snake grass (*Clinacanthus Nutans Lindau*) respectively. The mean of the expenditure on CAM usage was RM 52.8 ± 101.9 (US $16.9 ± 32.5) per month. According to multiple logistic regression analyses, being Muslim (OR 5.258, 95 percent CI 2.952-9.368) had significant positive association with CAM use.

**Conclusions:**

The prevalence of CAM use was high among diabetics. Islam faith is predictor for CAM use among Type 2 DM patients. The most-common herbs used were bitter gourd (*Momordica Charantia*) and Misai Kucing (*Orthosiphon Stamineus, Benth*). Further studies on the anti-glycemic activity of the isolated compound may be needed in the future.

## Background

Complementary and alternative medicine (CAM) is defined as a group of diverse medical and healthcare systems, practices, and products that are not generally considered part of conventional Western medicine [1,2]. CAM use can be divided into five categories: biological-based therapies like herbal and dietary supplement; alternative medical systems like acupuncture or Ayurveda; energy therapies like Reiki; manipulative and body-based systems like chiropractic or massage; and mind-body interventions like tai chi or yoga [3].

CAM usage is common among patients with diabetes mellitus (DM). A better understanding of CAM use will help the medical profession be more vigilant and patient-centered, particularly during counseling sessions regarding proper use herbal remedies in the stream of modern medicine.

The prevalence of CAM usage among DM patients has a wide range (17–72.8 percent) due to different definitions in the studies [4]. Studies show that white middle-aged, being women, receiving higher education and those suffer from more than one chronic disease (especially metabolic, mental, and musculoskeletal disorders) are having a higher correlation with the use of CAM [5,6].

Studies reported that herbal remedies, vitamins, spirituality, and exercise are common CAM therapies pursued by diabetics [4]. Among the herbal remedies, true cinnamon (*Cinnamomum verum*) is used commonly in the United States and Canada [7]. Bitter gourd (*Momordica Charantia*) and garlic (*Allium Sativum*) are predominantly used in India [8]. In Asia and Mediterranean, fenugreek (*Trigonella Foenum Graecum*) has been cultivated and used medicinally for thousands of years [9,10]. In Malaysia, there are 12,000 plants. However, only about 1,300 herbs have been found to have therapeutic benefits [11]. This indicates that local herbs were underutilized, as not much study has been done on useful local herbs in treating or controlling diabetes [12]. Studies actually show that CAM remedies can be an important component of health self-management, depending on the patient’s financial resources, culture, and self-empowerment [13,14]. Therefore, this study was conducted to examine the prevalence, types, reasons, and expenditures devoted to CAM among DM patients attending an outpatient primary-care clinic in Malaysia.

## Methods

### Setting

This is a cross-sectional study of patients registered with the primary health care clinic at Salak in Sepang, Selangor, Malaysia. This clinic is run by a family medicine specialist and 10 medical officers.

### Inclusion criteria

All registered diabetics more than age 18 and above and a minimum follow-up of three months at Klinik Kesihatan Salak, Sepang, were eligible for the study. The sample size was calculated by using Epi Info 6.0, based on the prevalence in local studies, which ranged from 50–56 percent [15,16]. The estimated sample size was 185 with 90 percent power, 95 percent confidence interval (CI), and statistical significant level (α) at 5 percent. The total number of respondents needed was 240, after taking into account a non-respondent rate of 30 percent. Patients were selected using a random sampling method. The estimated number of diabetic patients that visit the clinic per day and in three weeks’ time were 32 patients and 480 patients. Since the number of required test subjects was 240, the sampling interval of two was used as the constant difference between subjects. The first starting number of 2 was picked randomly from the registration counter.

### Data collection

A face-to-face interview was conducted using a structured questionnaire. A written informed consent for participation in the study was obtained from participants. The questionnaire was designed to capture patients’ socio-demographic data, co-morbidities, types of CAM used, resources consulted, and the total expenditure on CAM. The attitudes, beliefs, and perceptions towards CAM were explored. Documented most-recent results of glycosylated hemoglobin (HbA1C) and blood pressure (BP) tests from the preceding year were captured from the patients’ medical records. A pilot study involving 42 patients was done to pretest the questionnaire and estimate the likely response rate. The main survey was administered during the first three weeks of May 2011 by three medical students.

### Practical definition

A DM patient was defined as someone who was clinically diagnosed with diabetes or was taking diabetic medications. Patients with hypertension were those whose BP ≥ 140/90 mmHg or were on antihypertensive agents.

CAM use in this study is defined as consumption in one of the five categories therapy: biological-based therapies like herbal and dietary supplement; alternative medical systems, like acupuncture or Ayurveda; energy therapies like Reiki; manipulative and body-based systems like chiropractic or massage; and mind-body interventions like tai chi or yoga [3].

### Data analysis

Statistical Package for Social Sciences (SPSS) v. 19.0 was used to analyze the data collected from the study. The findings were described in terms of frequencies, percentages, means, and standard deviations. The association between socio-demographic factors (gender, age, race, religion, educational, occupation, family household income, and duration and control of diabetes) and the CAM usage was determined by using Chi-square test. Multivariate logistic regressions were used to identify predictors of CAM usage.

### Ethical approval

Ethical approval was obtained from the Ethics Committee of National Malaysia Research Registry (NMRR-12-430-11052).

## Results

### Socio-demographic characteristics

A total of 252 subjects was eligible in the original cohort; 12 of them refused to participate in the study. In the end, 240 diabetic patients were enrolled into the study with the response rate of 95 percent. Table 1 shows the socio-demographic information of respondents. The respondents were predominantly female Malayan Muslims 50–69 years old with a mean age of 55.14 ± 10 years. Most received primary education and the average monthly household income was RM 1843.17 ± 1537 (USD 588). The mean duration of diabetes and the mean HbA1c were 6.5 ± 5.7 years and 8.7 ± 2.8 percent respectively. Most respondents (72.9 percent) had underlying hypertension followed by dyslipidemia (11.3 percent), asthma (1.7 percent), and osteoarthritis (0.8 percent). The mean systolic and diastolic blood pressures were 138 ± 19 and 82 ± 12 mmHg, respectively.

**Table 1:** Demographic and clinical characteristics of the diabetic respondents in Klinik Kesihatan Salak (N=240)

**Socio-demographic factors**	**Total subject N=240, (%)**	**Using CAM n=150, (%)**
**Gender**		
Male	95(39.6)	54(36.0)
Female	145(60.4)	96 (64.0)
**Race**		
Malays	145 (60.4)	112 (74.7)
Chinese	14(5.8)	9 (6.0)
Indians	79(32.9)	27 (18.0)
**Religion**		
Muslim	150(62.5)	116 (77.3)
Buddhist	13(5.4)	8 (5.4)
Hindu	71(29.6)	24 (16.0)
Christian	6(2.5)	2 (1.3)
**Level of Education**		
No education	31(12.9)	18 (12.0)
Primary	99(41.2)	57 (38.0)
Secondary	94(39.2)	64 (42.7)
Tertiary	16(6.7)	11 (7.3)
**Occupation**		
Blue collar	101(42.1)	52 (34.7)
White collar	139(57.9)	98 (65.3)
**Family Household Income**		
0 – 2500	189(78.8)	116 (77.3)
2501 – 5000	44(18.3)	29 (19.3)
5001 – 7500	3(1.2)	1 (0.4)
7501–10000	4(1.7)	4 (1.7)

### Types of CAM used by DM patients

The prevalence of CAM use was 62.5 percent. Female were 1.8 times more likely than male. Malays (75%) were the most frequent users, followed Indians (18%) and Chinese (6%). Table 2 shows CAM use among DM patients. Biological therapy which involved the herbal products (50.0%) were the most widely used, followed by manipulative-body based systems (9.2%), energy system (8.8%), alternative medicine systems (4.6%) and mind-body system (1.7%). Bitter gourd (30.4 percent, n=73) was the most popular natural product consumed by respondents. Other commonly used herbal products included Misai Kuching (24.2 percent, n=58) and garlic (13.3 percent, n=32). Surprisingly, none of them sought help from a religion master and/or “bomoh.”

**Table 2:** Type of CAM used by DM patients in Klinik Kesihatan Salak (N=150)

**Type of CAM**	**Frequency**	**Percentage**
**Biological based therapy like Herbal products**	**120**	**80.0**
Bitter gourd	73	48.7
Misai Kuching	58	38.7
Garlic	32	21.3
Sabah snake grass	19	12.7
Basil leaf	5	3.3
Ginseng	4	2.7
**Manipulative and body-based systems**	**22**	**14.7**
Reflexology	22	14.7
**Alternative medical systems**	**11**	**7.3**
Ayurveda	10	6.7
Acupuncture	3	2.0
**Energy therapies**	**21**	**14.0**
Reiki	3	2.0
Massage bed	18	12.0
**Mind-body interventions**	**4**	**2.7**
Yoga/tai chi	4	2.7

### Attitudes, beliefs, and perceptions toward CAM

More than half of survey respondents pursued CAM therapies because they believed CAM can help them achieve better control in diabetes (58.0 percent) and better value for money (17.3 percent). Some use it because they are following the example of other CAM users (17.3 percent) (Table 3).

**Table 3:** Attitudes, beliefs, and perceptions questionnaires toward CAM (N=150)

**Attitudes, beliefs and perceptions towards complementary alternative medicine**	**n**	**%**
Believe CAM can help the diabetes control	87	58.0
Having good example from the other user of CAM and keen to share with others	26	17.3
Easily available and better value for money	26	17.3
Dissatisfied with western medicines	5	3.3
Use for other co morbidity treatment	5	3.3
Believed that CAM had fewer side effects	1	0.7

### Resources on CAM

This study found that most respondents learned about CAM primarily from friends (32.1 percent) and family (13.8 percent) followed by media (13.3 percent) and health professionals. The mean duration of CAM usage was 4.0 ± 4.6 years. The mean frequency of consumption was 3.5 times per week.

### Expenditures on CAM

The mean of the total out-of-pocket expenditure on CAM usage was RM 52.8 ± 101.9 (US $16.9 ± 32.5) per month. The vast majority (87.5 percent) of respondents spent RM 52.8 (US$16.9) or less per month on CAM. Thirty patients (12.5 percent) spent more than RM 52.8 (US$16.9) per month on CAM therapies.

### Multivariate logistic regression

Table 4 summarizes the characteristic differences between CAM users and non-CAM users. The results of unadjusted univariate logistic regression analysis of variables related to CAM users were calculated. A multivariate logistic regression analysis was used to independently predict a CAM user after adjustment for variables that attained P < 0.05 in univariate analysis and clinical significant variables. Being Muslim (odds ratio [OR] 5.258, 95 percent, CI 2.952-9.368) is the only predictor for CAM use after adjustments for gender, ethnicity, religion, occupation, family household income and hypertension.

**Table 4:** Association of characteristics between patients who used CAM and those who did not use CAM (N=240)

**Socio-demographic factors**	**No CAM use**	**CAM use**	
	**n=90, (%)**	**n=150, (%)**	**p-value**
**Gender**			
Male	41 (45.6)	54 (36.0)	0.163
Female	49 (54.4)	96 (64.0)	
**Ethnicity**			
Malays	33(36.7)	112 (74.7)	<0.001
Chinese	5 (5.6)	9 (6.0)	
Indians	52 (57.8)	27 (18)	
**Religion**			
Islam	34(37.8)	116 (77.3)	<0.001
Buddhist	5 (5.6)	8 (5.3)	
Hindu	47 (52.2)	24 (16.0)	
Christian	4 (4.4)	2 (1.3)	
**Education**			
Primary school and below	55 (61.1)	75(50.0)	0.115
Secondary school and above	35 (38.9)	75 (50.0)	
**Occupation**			
Blue collar	48(53.3)	52 (34.7)	0.005
Non Blue collar	42 (46.7)	98 (65.3)	
Hypertension	63 (70.0)	112 (74.7)	0.431
Family Household Income (n, SD)	1536±1236	2030±1666	0.019
HbA1c (n, SD)	8.5±1.7	8.9±3.0	0.448

There is significant association if p-value <0.05 *.

*CAM*: complementary alternative medicine.

*SD*: standard deviation.

*n*=number.

## Discussion

The prevalence of CAM usage among DM patients in this study population was high (62.5 percent). This is consistent with findings in other studies [4]. The current usage is higher than studies in the United Kingdom (17 percent) [17], Australia (23.6 percent) [18], Turkey (41.0 percent) [19], and Thailand (47 percent) [20]. This is comparable to studies in Taiwan (61 percent) [21] and Mexico (62 percent) [22] and lower compared to Korea (65 percent) [23], India (67.7 percent) [24], and the U.S. (72.8 percent) [25]. Our results were much higher compared to the local population study, where only 2.3 percent of Malaysians consumed CAM overall [26] and 0.2 percent of DM patients had experience in using CAM [27]. However, this may be underreported as other studies mention that patients with chronic diseases like diabetes tend to consume CAM compared to the general population [4,7,25,28,29]. Our result is still higher when compared to the two other local studies done at the Ipoh primary care clinic (56 percent) and Seremban Government Hospital diabetes health clinic (49.6 percent) respectively [15,16].

Previous studies reported that the reasons for DM patients to choose such therapies may be related to the fact that diabetes is a chronic, devastating, and incurable disease. Patients may have positive views of CAM due to its organic nature (which can present fewer side-effects), concerns about doctors’ listening skills, preferences to be treated holistically, and increased availability of CAM [10,30].

In the present study, only a small portion of patients were dissatisfied with conventional medicines (3.3 percent) and believed that traditional medicine presented more adverse effects (0.7 percent). This was surprising, since previous studies had shown dissatisfaction with conventional treatments due to ineffectiveness or unpleasant side effects were the common reasons for pursuing CAM. This is consistent with a US-based study that reported “users of alternative health care are no more dissatisfied with or distrustful of the conventional care than nonusers are” [10]. One possible reason for our findings is a change in society that links to a patient self-empowerment paradigm [31]. Most DM patients had poor blood-sugar control, as the conventional treatment requires them to be disciplined with respect to diet, lifestyle, and behavior [32,33]. Because of this, patients tend to try CAM to optimize their health status so that they feel that they have partially contributed to the management of their disease. They believe CAM offers more personal autonomy and control over their disease [34-36]. On the other hand, it could be related to underlying shared beliefs and cultural assumptions [37].

In our study, the main types of CAM used were herbal and dietary supplements as well as reflexology. The high consumption is not surprising, since most diabetics presumed that herbs are safer and, additionally, more affordable and easily available [16,38,39]. This was further supported by the fact that the total out-of-pocket expenditure was RM 52.8 ± 101.9 (US $17.0 ± USD32.8) per month. The most common sources of CAM information were recommendation from friends and families. The present study indicates that we must involve patients’ friends and the families during diabetes education counseling regarding the efficacy and potential side-effects of CAM.

Malaysia is a multiethnic, multicultural, multi-religious developing nation in which Malays form the majority, constituting 50.4 percent of the population [40]. CAM use is deeply rooted and influenced by its multicultural and religious nature. It has had ethnic diversity and this influences CAM use as well. Uses of herbs [41] like bitter gourd [42-44], Misai Kucing (*Orthosiphon Stamineus Benth*) [45,46], garlic (*Allium Sativum*) [47,48], and ginseng (*Panax Ginseng*) [49] are believed to reduce blood sugar levels. Bitter gourd [50], also known as *Momordica Charantia* is a tropical vine that is widely believed to bring down blood sugar levels, despite a lack of robust evidence [43,44,50-52]. Bitter gourd was widely used as ayuverda treatment in India. It was found to be the most common herb used as before 15^th^ century and traditional Malay medicine has been strongly influenced by the animistic culture of Hindu-Buddhism, thus the use of the bitter gourd is already deeply ingrained in the Malay population [26].

Interestingly, Sabah snake grass (*Clinacanthus Nutans Lindau*), an anti-inflammatory used for treating insect bites and herpes infections in Thailand [53], has also been widely used by DM patients. The users believe it contains anti-glycemic components. This may merit further study.

Belief in Islam was found to be one of the strongest predictors for pursuing CAM therapy. One explanation for this may be that CAM usage has always been embedded into the Muslim belief system and cultural heritage that is already deeply integrated into their lives [54,55]. Indeed, Malaysia is unique in its role as a confluence of three Asian cultures, giving rise to three main traditional healing practitioners. However, Malaysia is an Islamic country, so this may explain why only a belief in the Muslim religion is a predictor of CAM usage.

Older female patients with higher levels of education and household income were more likely to be CAM users in some studies [56-58]. However, the present study found no significant relationship in CAM usage and gender, mean age, ethnic group, education level, or total household income. This could be because the studied population was DM patients, who might be more likely to resort to CAM therapies - regardless of gender or socio-demographic status. This is supported by a U.S.-based study that reported that DM patients were 1.6 times more likely to use CAM than non-diabetics [58]. The HbA1C also was not found to have relationship as the control of diabetes was confounded by other factors such as compliance as well as other treatment modalities. This is important because, although there is increasing global interest in CAM use worldwide, doctors who practice Western medicine seem less aware about its significance and importance. By right, as health-care professionals, we should be knowledgeable about potential benefits and possible toxicities of such remedies [59]. Practitioners should provide evidence-based information on safety issues, efficacy, and potential interactions among commonly used CAM treatments – instead of brushing the topic aside or ignoring its usage.

### Strength and limitations

This will be interesting to include non-diabetic group as the control group in this study. However, it was not done due to the time constraint. Enquiry into the number of prescribed medication is important but not done in this study as this is not the primary objective. It is acknowledged that a comparison of rates of CAM use among DM patients across different studies is limited due to the differences in the definitions and inclusions/exclusions of CAM therapies in each study. However, these will not affect our findings in any way.

## Conclusions

This study showed that the prevalence of CAM consumption/use was higher among DM patients. The high consumption/use of CAM should prompt clinicians to further explore this topic, particularly among DM patients who are Muslim. In addition, future studies are recommended to conduct a randomization trial that analyzes these herbs particularly bitter gourd, Misai Kuching, garlic, and sabah snake grass – in reducing blood sugar levels in local settings.

## Abbreviations

CAM: Complementary alternative medicine; BP: Blood pressure; DM: Diabetes mellitus; SPSS: Statistical package for social sciences; HbA1C: Glycosylated hemoglobin; USD: United States dollar; RM: Ringgit Malaysia; OR: Odds ratio; CI: Confidence interval.

## Competing interests

The authors declare that they have no competing interests.

## Authors’ contributions

SMC and FP involved in the study design, data collection, performed the statistical analysis and drafted manuscript. ZAZ and MJ helped to design the study and drafting the manuscript. All authors read and approved the final manuscript.

## Pre-publication history

The pre-publication history for this paper can be accessed here:

http://www.biomedcentral.com/1472-6882/13/148/prepub
